# Regulatory Functions and Mechanisms of Circular RNAs in Hepatic Stellate Cell Activation and Liver Fibrosis

**DOI:** 10.3390/cells12030378

**Published:** 2023-01-19

**Authors:** Archittapon Nokkeaw, Pannathon Thamjamrassri, Pisit Tangkijvanich, Chaiyaboot Ariyachet

**Affiliations:** 1 Department of Biochemistry, Faculty of Medicine, Chulalongkorn University, Bangkok 10330, Thailand; 2 Center of Excellence in Hepatitis and Liver Cancer, Faculty of Medicine, Chulalongkorn University, Bangkok 10330, Thailand; 3 Medical Biochemistry Program, Department of Biochemistry, Faculty of Medicine, Chulalongkorn University, Bangkok 10330, Thailand

**Keywords:** non-coding RNA, circular RNA, microRNA, hepatic stellate cells, liver fibrosis

## Abstract

Chronic liver injury induces the activation of hepatic stellate cells (HSCs) into myofibroblasts, which produce excessive amounts of extracellular matrix (ECM), resulting in tissue fibrosis. If the injury persists, these fibrous scars could be permanent and disrupt liver architecture and function. Currently, effective anti-fibrotic therapies are lacking; hence, understanding molecular mechanisms that control HSC activation could hold a key to the development of new treatments. Recently, emerging studies have revealed roles of circular RNAs (circRNAs), a class of non-coding RNAs that was initially assumed to be the result of splicing errors, as new regulators in HSC activation. These circRNAs can modulate the activity of microRNAs (miRNAs) and their interacting protein partners involved in regulating fibrogenic signaling cascades. In this review, we will summarize the current knowledge of this class of non-coding RNAs for their molecular function in HSC activation and liver fibrosis progression.

## 1. Introduction

Chronic liver disease (CLD) represents a gradual deterioration of liver functions, which results from a spectrum of aetiologies including toxins, viral hepatitis, alcohol abuse, and non-alcoholic fatty liver disease (NAFLD) [1]. CLD involves a replacement of healthy liver tissues with fibrotic scars, and hepatic stellate cells (HSCs) play a major role in this process at a cellular level. HSCs are a liver mesenchymal cell type residing in Disse space. In normal livers, HSCs are quiescent and store retinol in cytoplasmic lipid droplets, but upon liver injury, HSCs become activated, lose retinol storage, and trans-differentiate into myofibroblasts that produce extracellular matrix (ECM) mainly type I collagen [2,3]. Excessive ECM accumulation is one of the critical hallmarks of liver fibrosis, disrupting liver architecture and forming fibrous scars [4]. For a short-term injury, fibrosis could be reversed by HSC inactivation and/or apoptosis [5]. However, persistence of injury promotes the continuous production of ECM and eventually leads to irreversible liver fibrosis and cirrhosis [6]. Cirrhosis is the end-stage CLD where the liver structures and functions are severely compromised [7,8].

Several signals play essential roles in HSC activation, such as damage-associated molecular patterns (DAMPs) [9], transforming growth factor beta (TGF-β) [10], platelet-derived growth factor (PDGF) [11], NLRP3 inflammasome [9], and WNT/β-catenin [12]. Recent studies reveal the roles of non-coding RNAs (ncRNAs) in controlling these signals and that modulation of these ncRNAs can either accelerate or inhibit fibrosis progression [13]. For example, microRNAs (miRNAs) including miR-155, miR-150-5p, and miR-130b-5p are reported as liver fibrosis promoters [14,15,16]. On the other hand, miR-130a-3p, miR-29a, and miR-101 are found to be anti-fibrotic factors [17,18,19]. In addition, long non-coding RNAs (lncRNAs) such as HOXA distal transcript antisense RNA (HOTTIP), HOX antisense intergenic RNA (HOTAIR), and hepatocellular carcinoma upregulated lncRNA (HULC) have been identified as regulators of HSC activation [20,21,22,23]. Recently, a novel type of ncRNAs, circular RNAs (circRNAs), has been found to modulate liver fibrosis by regulating the activity of miRNA targets or interacting with RNA-binding proteins (RBPs) [24,25]. In this review, we will focus on the roles of circRNAs and their molecular mechanisms in HSC activation, which causes the development and progression of liver fibrosis.

## 2. Non-Coding RNA (ncRNA)

Only approximately 1.5% of our genome encodes 21,000 proteins via the central dogma [26,27]. The remaining genomic DNA are non-coding, but a large portion can be transcribed into RNA strands, known as non-coding RNAs (ncRNAs) [26]. Unlike mRNAs, ncRNAs do not translate into proteins but can function as structural, catalytic, and regulatory RNAs [28,29]. Several types of ncRNAs assist gene expression such as small nuclear RNAs (snRNAs), transfer RNAs (tRNAs), and ribosomal RNAs (rRNAs) [27]. Other ncRNAs including microRNAs (miRNAs), long non-coding RNAs (lncRNAs), and circular RNA (circRNAs), play a crucial role in epigenetic mechanisms [25,30,31].

## 3. Circular RNAs (circRNAs)

CircRNAs, a newly discovered type of ncRNAs, are single-stranded, covalently closed RNAs without 3′ poly(A) tails and 5′ end caps and were first identified in RNA viruses as viroids [32]. CircRNAs are previously believed to be the consequence of splicing errors [33]. However, accumulating evidence show that many circRNAs are highly conserved across species with differential expression patterns in developmental stages and physiological conditions, indicating their potential roles in biological processes [34,35]. Due to their circular structure, which lacks free ends, circRNAs are more stable than linear mRNAs. Most mRNAs exhibit an average half-life of approximately 9 h [36], while the half-life of circRNAs can reach 48 h [37]. This prolonged lifespan of circRNAs may be due to their resistance to degradation by exonucleases [38].

CircRNAs could consist of a single exon or, more often, several exons of protein-coding sequences; this class of circRNAs is known as exonic circRNAs (ecircRNAs) [39]. Some circRNAs might contain only introns instead of exons; these types of circRNAs are called circular intronic RNAs (ciRNAs) [40]. Furthermore, some circRNAs may include both introns and exons which are known as exon-intron circRNAs (EIciRNAs) [41]. Interestingly, circRNAs can also derive from the intron region of a tRNA gene. These circRNAs are called tRNA intronic circular RNAs (tricRNAs) [42].

## 4. Biogenesis and Transportation of circRNAs

CircRNAs are often formed via back-splicing of precursor mRNAs (pre-mRNAs) in which a downstream 3′ splice-donor is covalently connected to an upstream 5′ splice-acceptor. CircRNAs may also be formed through splicing intermediates called lariat precursors of pre-mRNAs resulting from exon-skipping or intronic lariat precursors that escape the debranching [43,44]. Back-splicing is naturally unfavorable [45]; consequently, most circRNAs are less abundant than their linear mRNA isoforms [46]. Nevertheless, cells possess *cis*- and *trans*-acting elements to facilitate back-splicing by bringing the downstream splice-donor site and the upstream splice-acceptor site in proximity [45,47,48]. Particularly, reverse complementary sequences (such as *Alu* elements) play crucial roles in the formation of circRNAs in a process called intron-pairing-driven circularization [34,37,49]. Moreover, RNA-binding proteins (RBPs) can act as *trans*-acting elements to promote circularization by bringing the splice sites within proximity of each other or stabilizing intronic RNA pairs [50,51]. These RBPs include muscleblind (MBL) [50], quaking (QKI) [52], heterogeneous nuclear ribonucleoprotein L (HNRNPL) [53], nuclear factor 90/110 (NF90/NF110) [51] and fused in sarcoma (FUS) [54]. Some *trans*-acting elements, for instance, DEAH (Asp-Glu-Ala-His) box helicase 9 (DHX9) and adenosine deaminase acting on RNA 1 (ADAR1), have been reported to decrease circRNA biogenesis by disrupting base pairing between flanking inverted repeat elements [34,55,56]. Intron-pairing-driven and/or RBP-mediated circularization results in the formation of EIciRNAs or ecircRNAs if introns are further removed [57]. Some circRNAs can be formed via lariat-driven circularization [37,44], enabling the formation of EIciRNAs, which can further undergo internal splicing to remove the intronic sequence and generate ecircRNAs [41,44]. Moreover, lariat-driven circularization can also promote ciRNAs generation [58].

Except for those with introns (EIciRNAs and ciRNAs), most circRNAs are transported to the cytoplasm after biogenesis [41,59,60]. ATP-dependent RNA helicase DDX39A (URH49) and spliceosome RNA helicase DDX39B (UAP56) play key roles in this process to export circRNAs with a small and large size, respectively, from the nucleus to the cytoplasm [61].

## 5. Degradation of circRNAs

Unlike mechanisms of circRNA biogenesis, details of how circRNAs are degraded in cells remain largely unknown. CircRNAs are resistant to exonuclease catalysis because they lack free 5′ or 3′ ends, so endonuclease is required for the degradation process [62]. RNase L, an endoribonuclease, is identified as a potential enzyme that can globally degrade circRNAs [63]. Moreover, other mechanisms in the degradation of circRNAs have been reported, including (i) Argonaute 2 (AGO2)-mediated cleavage [64], (ii) degradation by the RNase P/mitochondrial RNA processing (MRP) Complex [59], (iii) the up-frameshift suppressor 1 homolog (UPF1)/endo-ribonuclease Ras GTPase-activating protein-binding protein 1 (G3BP1)-mediated RNA decay [65], (iv) RNase H1-mediated degradation [66], (v) TMAO-mediated degradation [67], and (vi) exosome-mediated degradation [68]. These mechanisms are considered to be able to decrease the levels of circRNAs.

## 6. Role of circRNAs

CircRNAs serve an essential non-coding function in various cellular processes. Other than competing with pre-mRNA splicing and lowering the levels of linear mRNA isoforms, leading to altered gene expression [43,47,50], some speculate that they act as miRNA sponges if they contain multiple miRNA binding sites [69,70,71,72]. Moreover, since circRNAs possess protein binding sites [39], circRNAs can affect cellular function by controlling activity of their protein partners [50,73,74]. Despite the lack of a 5′ cap structure and a 3′ Poly(A) tail, circRNAs can be translated into proteins via the internal ribosome entry site (IRES) and *N*^6^-methyladenosines (m^6^A)-mediated cap-independent translation [75,76].

### 6.1. Acting as miRNA Sponges

**Figure 1:**
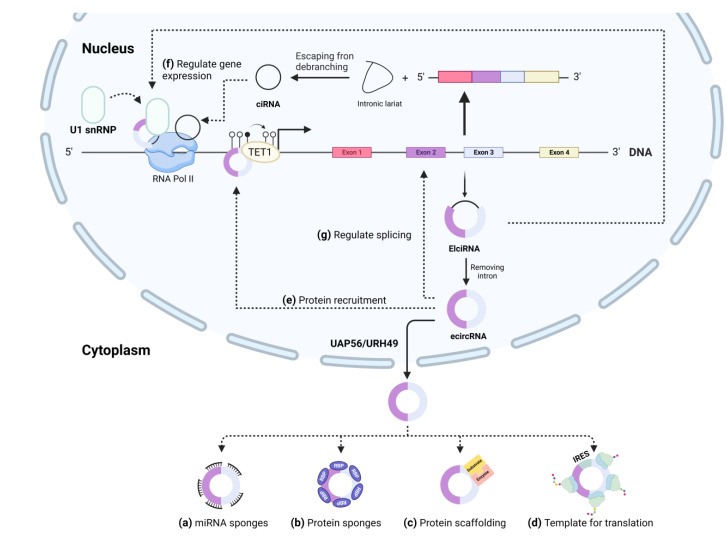
Biological roles of circRNAs. (**a**) Circular RNAs (circRNAs) can function as microRNA (miRNA) sponges, promoting their degradation and increasing the expression of target mRNAs. (**b**) CircRNAs with RNA-binding protein (RBP) binding sites have the potential to decoy these proteins and disrupt their functions. (**c**) Some circRNAs have been demonstrated to serve as protein scaffolds, allowing the colocalization of proteins (e.g., enzymes and their substrates). (**d**) CircRNAs containing an internal ribosome entry site (IRES) or N^6^-methyladenosine (m^6^A) and a start codon can be translated to proteins under certain conditions. (**e**) Additionally, circRNAs may attract certain proteins to a particular location in the cell. (**f**) CircRNAs can alter gene expression by interacting with RNA polymerase II (RNA Pol II). (**g**) Some circRNAs can affect the splicing of the host gene and influence the ratio between mRNAs and circRNAs. Abbreviation: Tet1: tet methylcytosine dioxygenase 1; U1 snRNP: U1 small nuclear ribonucleoprotein particle; EIcircRNA: exon-intron circRNAs; ecircRNA: exonic circRNA; ciRNA: circular intronic RNAs; UAP56: spliceosome RNA helicase DDX39B; URH49: ATP-dependent RNA helicase DDX39A.

MiRNAs are a class of small and highly conserved non-coding RNA molecules that can regulate gene expression [77]. Most miRNAs are transcribed from DNA into primary miRNAs (pri-miRNAs) by RNA polymerases II and III [78]. Then, a class 2 ribonuclease enzyme process these pri-miRNAs into precursor miRNAs (pre-miRNAs), following by a cascade of cleavage steps to generate mature miRNAs [78,79]. Then, a mature miRNA duplex is incorporated into the AGO protein family to form the miRNA-induced silencing complex (miRISC) that can target mRNAs and cause mRNA degradation and/or translational repression [77,78,79,80,81], resulting in altered gene expression. CircRNAs appear to play an important role in the miRNA-mediated regulation of gene expression, possibly by sponging specific miRNAs and thus increasing protein levels of miRNA target genes (Figure 1a) [82,83,84,85]. Nevertheless, a caution should be taken that several miRNA binding sites within a circRNA are required for efficient sequestration of a particular miRNA [86].

### 6.2. Interacting with Proteins

CircRNAs may bind with RBPs via RBP-binding sites [50]. RBPs play essential roles in the control of gene expression by regulating splicing, mRNA maturation, transport, localization, translation, and decay [87,88,89]. Various circRNAs have been discovered to interact with RBPs by acting as protein sponges or decoys, thus activating, or disrupting their biological functions (Figure 1b) [90,91,92]. Some circRNAs serve as a protein scaffold to promote the colocalization of two or more proteins, such as enzymes and their substrates, enhancing their activity (Figure 1c) [74,93]. Some circRNAs can recruit certain proteins to specific cellular locations and spatially modulate their function **(**Figure 1e) [94,95,96]. EIciRNAs and ciRNAs, which are located in the nucleus, are involved in transcriptional regulation by promoting RNA Pol II activity, which enhances target gene transcription (Figure 1f) [40,41]. Moreover, it has been revealed that some nuclear retained ecircRNAs influence splicing by exon-skipping variants, consequently affecting the balance between circRNAs and their linear isoforms (Figure 1g) [97].

### 6.3. Translated into Proteins

Linear mRNA translation is usually a cap-dependent process that requires a 7-methylguanosine (m^7^G) cap at the 5′ end and a poly(A) tail at the 3′ end [98]. Recent findings suggest that circRNA translation can occur in a cap-independent manner through IRES or m^6^A RNA modification in the 5′ untranslated region (UTR) (Figure 1d) [99,100]. CircMbl-encoded proteins are expressed in small amounts under normal conditions, but following starvation, their expression notably increases [101]. These results suggest that circRNA-derived peptides may play a role in maintaining homeostasis.

## 7. CircRNAs and Hepatic Stellate Cell Activation

**Figure 2:**
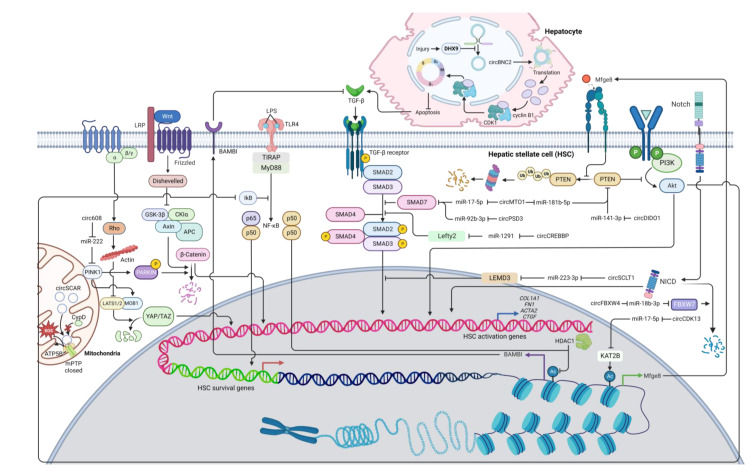
A diagram illustrating the regulatory networks of HSC activation modulated by anti-fibrotic circRNAs. Arrows represent activation, whereas bars symbolize inhibition. Abbreviation: *COL1A1*: collagen type I alpha 1 chain; CypD: Cyclophilin D; FBXW7: F-box/WD repeat-containing protein 7; HLF: hepatic leukemia factor; KAT2B: lysine acetyltransferase 2B; LEMD3: LEM domain containing 3; Lefty2: left-right determination factor 2; mPTP: mitochondrial permeability transition pore; PINK1: PTEN-induced kinase 1; PTEN: phosphatase and tensin homolog; TGF-β: transforming growth factor beta; TLR4: toll-like receptor 4.

**Figure 3:**
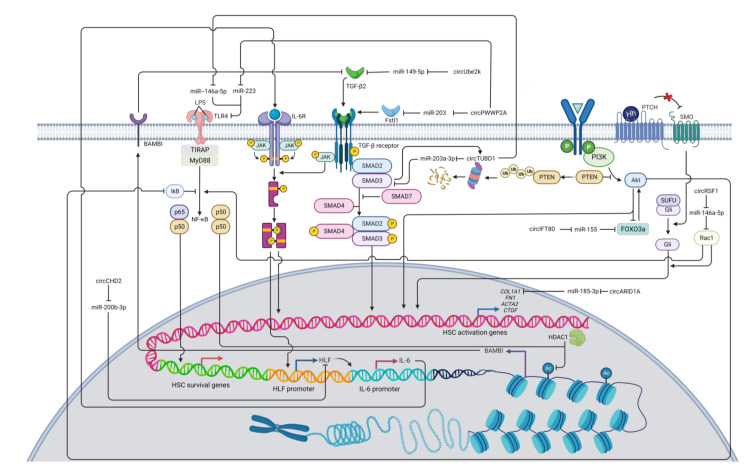
A diagram illustrating the regulatory networks of HSC activation modulated by pro-fibrotic circRNAs. Arrows represent activation, whereas bars symbolize inhibition. Abbreviation: *COL1A1*: collagen type I alpha 1 chain; HLF: hepatic leukemia factor; HDAC1: histone deacetylase 1; IL-6: interleukin 6; PTEN: phosphatase and tensin homolog; RAC1: Ras-related C3 botulinum toxin substrate 1; TGF-β: transforming growth factor beta; TLR4: toll-like receptor 4.

HSC activation is associated with several cytokines and regulatory networks and promotes expression of HSC activation markers, including α-smooth muscle actin (α-SMA) and type I collagen [102]. Many signaling pathways have been linked to increasing expression levels of α-SMA and/or type I collagen, including TGF-β [103], JAK/STAT [104], PDGF [105], PI3K/Akt [106], Wnt/β-catenin [107], Notch [108], Hedgehog [109,110], Hippo [111], and inflammasome signaling pathways [112]. Thus, circRNAs targeting these signaling pathways can regulate HSC activation and ultimately affect liver fibrosis (Table 1, Figure 2 and Figure 3).

### 7.1. Anti-Fibrotic circRNAs

TGF-β signaling is regarded as the primary fibrogenic pathway that stimulates HSC activation and ECM synthesis [103]. TGF-β is found in trace amounts in healthy livers [133]. Following liver damage, macrophages start producing TGF-β and PDGF, which can activate excessive ECM production from HSCs and lead to the development of liver fibrosis [134]. Most reported circRNAs involved in HSC activation target miRNAs and proteins in the TGF-β pathway. One of these circRNAs is circPSD3 (mmu_circ_0001682), which is downregulated in primary HSCs and liver tissues from mice with carbon tetrachloride (CCl_4_)-induced liver fibrosis [113]. CircPSD3 can function as a miR-92b-3p sponge, consequently promoting Smad7 expression [113]. Smad7 can block the activation of receptor-regulated Smads (R-Smads) and thus inhibits the TGF-β signaling pathway [135,136]. Furthermore, circPSD3 can preclude HSC proliferation and forestall fibrosis progression in vivo [113].

CircCREBBP acts as a miR-1291 sponge, consequently increasing the expression of left-right determination factor 2 (LEFTY2) [114], which can inhibit the phosphorylation of Smad2/3 [137,138], a key signaling molecule in the TGF-β pathway [139]. Moreover, circCREBBP can also inhibit cell proliferation and arrest the cell cycle in HSCs [114]. With a similar mode of action, hsa_circ_0070963, also known as circSCLT1, has the ability to sponge miR-223-3p, which can target LEM domain containing 3 (LEMD3) [115], an inhibitory molecule that can antagonize Smad2/3 signaling and perturb the TGF-β signaling pathway [140]. In addition to suppressing HSC activation, the overexpression of circSCLT1 can induce cell cycle arrest and suppress cell proliferation in HSCs [115]. However, the circSCLT1/miR-223 axis is subject to further investigation as most studies suggest the anti-fibrotic roles of miR-223 in various fibrosis models [141,142,143,144]. Another circRNA is mmu_circ_34116, which is shown to suppress HSC activation upon overexpression [145]. Using bioinformatic techniques, mmu_circ_34116 is predicted to target the miR-22-3p/BMP7 axis [145]. Bone morphogenetic protein 7 (BMP7) can activate Smad1/5/8 but inhibits Smad3 activation, thus antagonizing TGF-β signaling [146,147].

CircMTO1 has been extensively studied for their anti-fibrotic roles. Its expression levels are observed to downregulate in liver tissues of fibrosis patients [116]. Patients with higher liver fibrosis stages have significantly less circMTO1 expression [116]. By interacting with miR-17-5p, circMTO1 can positively regulate the expression of Smad7 and thereby negatively regulate the TGF-β signaling pathway [116]. Moreover, circMTO1 is found to act as a miR-181b-5p sponge [117]. miR-181b-5p can target phosphatase and tensin homolog (PTEN), a negative regulator in the phosphatidylinositol 3-kinase/protein kinase B (PI3K/Akt) signaling pathway [117], which promotes HSC activation [106,148]. Therefore, circMTO1 plays an anti-fibrotic role by downregulating miR-181b-5p and enhancing PTEN activity [117]. Additionally, Akt signaling can also lead to the activation of NF-κB [149], protecting activated HSCs against apoptosis and sustaining cell survival [150]. As a consequence, by suppressing PI3K/Akt signaling via PTEN, circMTO1 overexpression potentially enhances HSC apoptosis and diminishes ECM production.

Some circRNAs play a significant role in other liver cell types but can indirectly have an impact on HSC activation. For example, in normal hepatocytes, circBNC2 expression is high but drastically decreased upon liver injury [118]. Interestingly, expression of activation markers (α-SMA and type I collagen) is significantly increased in HSCs incubated with conditional medium from circBNC2 knockout hepatocytes, which appear to contain high levels of the pro-fibrotic cytokines including TGF-β [118]. In contrast, the overexpression of circBNC2 in hepatocytes can reduce expression levels of these cytokines upon liver injury, and conditional medium from these hepatocytes can suppress expression of α-SMA and type I collagen in cultured HSCs, indicating the anti-fibrotic roles of circBNC2 [118]. Mechanistically, circBNC2 contains an open reading frame (ORF) and an IRES, suggesting their function as a protein template [118]. The circBNC2-translated protein (ctBNC2) is a protein product derived from circBNC2 translation [118]. This 681-amino acid protein can bind to CDK1 and cyclin B1 and promote CDK/cyclin complex translocation into the nucleus, a critical step to initiate mitosis and prevent apoptosis [118]. As ctBNC2 levels decrease in a damaged liver, the translocation of the CDK/cyclin complex could be impaired and induce apoptosis, which triggers the secretion of TGF-β and DAMPs from hepatocytes [151,152]. As previously mentioned, these molecules can activate ECM production from HSCs and promote fibrosis progression. Therefore, by protecting hepatocytes from apoptosis upon injury, circBNC2 could play an anti-fibrotic role by suppressing production of inflammatory cytokines [118].

Another key signaling pathway that involves HSC activation is the network of PTEN/PI3K/Akt [106]. PI3K/Akt signaling can activate the serine/threonine kinase p70 ribosomal protein S6 kinase (p70S6K) via the mammalian target of rapamycin complex 1 (mTORC1), whereas PTEN inhibits activation of PI3K/Akt signaling [106,153]. Evidently, p70S6K facilitates HSC proliferation and collagen expression [153], and thus, the PI3K/Akt signaling pathway is considered pro-fibrotic [106]. A recent report shows that circDIDO1 inhibits HSC activation through the miR-141-3p/PTEN axis [124]. The overexpression of circDIDO1 sponges miR-141-3p and increases PTEN expression, which impairs PI3K/Akt signaling and subsequently suppresses HSC activation [124]. Similarly, circCDK13 forestalls liver fibrosis by repressing the activation and proliferation of HSCs via the miR-17-5p/KAT2B/MFGE8/PTEN axis [125]. By sponging miR-17-5p, circCDK13 can promote the expression of lysine acetyltransferase 2B (KAT2B), a protein that can regulate histone protein acetylation [125]. The acetylation of histones can enhance the accessibility of transcriptional factors to DNA templates, thereby increasing transcriptional activity [154]. Specifically, KAT2B facilitates milk fat globulin-epidermal growth factor 8 (MFGE8) transcription by promoting histone H3 acetylation [125]. Because MFGE8 can inhibit PTEN ubiquitination and degradation [155], KAT2B promotes PTEN stability, which in turn suppresses the PI3K/Akt signaling pathway [125]. Inactivation of Akt can also lead to the reduction of NF-κB activity, thus impairing the survival of fibrogenic HSCs [125,156]. Collectively, circDIDO1 and circCDK13 can exert the anti-fibrotic mechanism via suppression of PI3K/Akt signaling.

In addition to TGF-β and PI3K/Akt signaling pathways, Wnt/β-catenin and hippo signaling could be regulated by circRNAs in HSCs. For example, circ608 increases PTEN-induced kinase 1 (PINK1) expression through sponging miR-222 [127]. PINK1 can then phosphorylate Parkin, an E3 ubiquitin-protein ligase, which in turn triggers ubiquitination and degradation of β-catenin, thereby inhibiting Wnt/β-catenin transduction [157,158]. PINK1 can also promote expression of Mps one binder kinase activator 1B (MOB1B) [159], a regulator of large tumor suppressor kinases 1/2 (LATS1/2) [160]. The MOB1/LATS complex can inactivate YAP and TAZ [160], the effectors in the Hippo signaling pathway [161], therefore inhibiting the Hippo signaling cascade and subsequently HSC activation. PINK1/Parkin can promote mitophagy [159], which is suppressed in fibrogenic HSCs [162]. Taken together, circ608 is an anti-fibrotic mediator via PINK1-mediated suppression of Wnt/β-catenin and hippo signaling.

For the Notch signaling pathway, circFBXW4 positively regulates F-box/WD repeat-containing protein 7 (FBXW7) [128], which induces Notch intracellular domain (NICD) degradation [163]. By interacting with miR-18b-3p, circFBXW4 can upregulate FBXW7 expression and inhibit Notch signaling, thereby inhibiting HSC activation and proliferation as well as promoting apoptosis [128].

A mitochondrial circRNA called circSCAR is found to be downregulated in NASH patients [130]. Lipid accumulation can induce endoplasmic reticulum (ER) stress, thus increasing the expression of the ER stress mediator CCAAT-enhancer-binding protein homologous protein (CHOP) [130]. CHOP can inhibit the expression of PGC-1α, which positively regulates circSCAR transcription and thus decreases circSCAR levels [130]. CircSCAR can interact with ATP5B, a regulator of the mitochondrial permeability transition pore (mPTP) [130]. Binding between circSCAR and ATP5B hinders the opening of mPTP and consequently the efflux of reactive oxygen species (ROS) from mitochondria into the cytosol [130]. Previous studies suggest that ROS promotes NF-κB signaling pathway and HSC activation [164,165]. Therefore, circSCAR may play an anti-fibrotic role by preventing leakage of ROS from mitochondria [130].

Some circRNAs are found to suppress HSC activation, but their underlying mechanisms are not fully understood. One example is hsa_circ_0004018 or circSMYD4, which is proposed to suppress HSC activation and proliferation via miR-660-3p [132]. By using bioinformatics tools, telomerase-associated protein 1 (TEP1) is predicted to be a target of miR-660-3p and experimentally confirmed by a luciferase assay [132]. While functions of TEP1 in HSCs have not been reported, higher levels of TEP1 in hepatocytes can indirectly suppress the proliferation and activation of HSCs, potentially because TEP1 prevents formation of critically short telomeres and reduces DNA-damage response, which normally triggers hepatocyte senescence and sends apoptotic signals to activate HSCs [132,166]. Intriguingly, although TEP1 play a role in telomere length regulation in many cell types [167,168], a study shows that TEP1 is not essential for telomere length regulation in murine liver [169]. This finding suggests the roles of TEP1 in other cellular functions that could modulate HSC activation [169]. In addition to being a component of the telomerase complex, TEP1 has been found in vault ribonucleoprotein complexes (VRCs) [170]. Although the function of VRCs in HSC activation is not yet known, major vault protein knock-out mice can intensify hepatic steatosis and induce fibrotic responses [171]. Further investigations are needed to expand the knowledge of how the circSMYD4/miR-660-3p/TEP1 axis inhibits HSC activation. Other examples are hsa_circ_0089761 and hsa_circ_0089763, which are two mitochondrial-encoded circRNAs downregulated in HSCs during LPS stimulation [172]. Their downregulation indicates their potential roles as anti-fibrotic circRNAs, but their mechanisms and targets remain to be further studied. Lastly, in a hepatitis B virus (HBV)-induced activation model of LX-2 cells, circMTM1 is upregulated along with interleukin 7 receptor (IL7R), potentially via absorbing miR-122-5p [173]. This study shows that circMTM1 knockdown expression and miR-122-5p overexpression reduce the expression levels of activated HSC markers, while the upregulation of IL7R attenuates the anti-fibrotic function of miR-122-5p [173]. Normally, IL7R is expressed in T cells and plays a pivotal role in T cell homeostasis [174]. Despite interesting results, the exact roles of circMTM1 and IL7R in HSC functions need a further investigation.

### 7.2. Pro-Fibrotic circRNAs

While most reported circRNAs impedes the activation of TGF-β signaling pathway in HSCs, some circRNAs play the opposite roles. These circRNAs include circPWWP2A [119], circUBE2k [120], and circTUBD1 [121,122]. CircPWWP2A can enhance TGF-β signaling by decreasing miR-203 and miR-223 expression and thus increasing expression levels of follistatin-like 1 (Fstl1) and Toll-like receptor 4 (TLR4), respectively [119]. Fstl1 is a glycoprotein ligand and can promote TGF-β signaling by binding to TGF-β1 type II receptors (TβRII) [175,176]. TLR4 can recognize lipopolysaccharide (LPS) and sequentially trigger signaling cascades that produce the NF-κBp50 homodimer to interact with histone deacetylase 1 (HDAC1). This protein complex can transcriptionally suppress BMP and activin membrane-bound inhibitor (BAMBI), a pseudoreceptor of TGF-β [119,177,178]. Since BAMBI can inhibit TGF-β/Smad signaling [177], TLR4-mediated downregulation of BAMBI can induce fibrotic HSCs via amplification of TGF-β signaling [119]. In addition, TLR4 can activate NF-κB to support the survival of the activated HSCs [179,180]. Together, circPWWP2A can promote HSC activation via sponging miRNAs that suppress the expression of Fstl1 and TLR4.

The overexpression of circUBE2K induces liver fibrosis by sponging miR-149-5p, which negatively regulates expression of TGF-β2 [120]. Conversely, inhibiting circUBE2K expression can impair TGF-β signaling and induce cell cycle arrest in HSCs, confirming the pro-fibrotic roles of circUBE2K in liver fibrosis progression [120]. Finally, circTUBD1 can interact with both miR-203a-3p and miR-146a-5p [121,122]. By sponging miR-203a-3p, circTUBD1 can positively regulate TGF-β signaling by increasing Smad3 expression [121]. Moreover, Smad3 is found to positively regulate circTUBD1 via a feedback loop, thus further enhancing Smad3 expression [121]. TLR4 is targeted by miR-146a-5p, and by downregulating miR-146a-5p via the circTUBD1 sponge, expression of TLR4 increases, thus promoting TGF-β and NF-κB signaling pathways, which can help activate and support cell survival of HSCs, respectively [122,177,179,180,181]. Furthermore, circTUBD1 can also promote HSC proliferation and inhibit the apoptosis of HSCs by increasing the expression of the anti-apoptotic protein B-cell lymphoma-2 (BCL-2), thereby amplifying the population of fibrogenic cells [122].

Interleukin-6 (IL-6) can activate HSC through the MAPK and JAK/STAT signaling pathways [104]. CircCHD2 can enhance hepatic leukemia factor (HLF) expression by interacting with miR-200b-3p [123]. HLF can promote the expression of IL-6 that can bind to the IL-6 receptor and activate the JAK/STAT3 pathway [182]. The JAK/STAT pathway is also a part of a non-SMAD pathway of TGF-β signal transduction that is essential for HSC activation [183]. The JAK/STAT3 pathway also promotes HLF expression, thereby regulating signal transduction in a feed-forward circuit manner [182].

Additionally, hsa_circ_0071410 (circPALLD) and hsa-circ-0067835 (circIFT80) are pro-fibrotic circRNAs that promote the PI3K/Akt signaling pathway [126,184,185]. CircPALLD can induce HSC activation and increase cell viability by interacting with miR-9-5p [184], which is reported to target annexin A2 (ANXA2) [185]. ANXA2 has been considered to play a role in HSC activation, proliferation, and apoptosis [185], possibly by activating the FAK/PI3K/Akt signaling pathway [186]. CircIFT80 can activate HSC by regulating the miR-155/Forkhead box O3 (FOXO3)/Akt axis [126,187]. As CircIFT80 alleviates FOXO3 expression by sponging miR-155, this transcription factor can transactivate PI3K/Akt through a positive feedback loop and promote PI3K/Akt signal transduction in fibrogenic HSCs [188,189].

The Hedgehog signaling pathway plays a significant role in HSC activation [109,110]. A recent study shows that circRSF1 sponges miR-146a-5p. Subsequently, this sequestration promotes Ras-related C3 botulinum toxin substrate 1 (RAC1) expression and Hedgehog signal transduction, resulting in HSC activation and proliferation [129]. Rac1 has been shown to be involved in the Hedgehog signaling pathway [190]. Activated Rac1 induces glioma-associated oncogene (Gli) nuclear translocation, which is necessary for the Hedgehog signaling pathway [191]. Rac1 can also influence NF-κB and JNK signaling by promoting their transduction [192,193]. JNK signal transduction can phosphorylate Smad2 at the C-terminal and linker regions [194]. Initial findings reveal that phosphorylation of Smad2 in the linker region hinders its nuclear translocation and cellular signaling, but recent evidence shows that phosphorylation of the Smad linker can stimulate expression of fibrotic genes [194,195]. One study discovers that the phosphorylated Smad2 linker region is associated with increased expression of glycosaminoglycans (GAG) [196], and hyaluronan (HA), a class of GAG, is found to be able to activate HSCs via Notch1 [197], so the phosphorylation of the Smad2 linker region may be associated with HSC activation by increasing the expression of HA and promoting the Notch1 fibrogenic signaling pathway. However, this proposed mechanism of circRSF1/miR-146a-5p/Rac1 with subsequent signal transductions remains to be further explored.

Moreover, the direct regulation of circRNAs on fibrotic genes have been reported. For instance, the circARID1A/miR-185-3p axis post-transcriptionally regulates expression of *COL1A1*, a key marker of HSC activation [131]. The pro-fibrotic circARID1A can also promote the proliferation and migration of HSCs as well as inhibit their apoptosis [131]. Mechanistically, increased type I collagen expression can lead to a positive feedback loop of HSC activation in which accumulation of collagen further activates HSCs by increasing ECM stiffness [198]. This mechanical tension in turn leads to YAP activation in the Hippo signaling pathway and Akt activation in the PI3K/Akt signaling pathway [198,199].

The above discussion mainly focuses on the intrinsic expression of circRNAs in HSCs as most *in vitro* studies rely on the single cell type for their analysis. However, apart from HSCs, liver is a complex organ comprising of several cell types including hepatocytes, Kupffer cells, liver sinusoidal endothelial cells, and cholangiocytes, which are known to communicate with one another to maintain liver functions [200]. Some of these cells were reported to modulate liver fibrosis by transferring circRNAs to HSCs such as hepatocyte-derived circBNC2 [118]. Alternatively, circRNAs that regulate the production of inflammatory cytokines in Kupffer cells can have an indirect impact on the state of HSC activation. These circRNAs include circMcph1 [201,202] and circ1639 [203,204,205,206]. Nevertheless, a study of cell-cell interaction in mediating liver fibrosis through circRNAs is still lacking and need to be further investigated.

## 8. Conclusions and Future Perspectives

HSC activation is the major contribution to liver fibrosis, which can trigger the development of cirrhosis and ultimately hepatocellular carcinoma (HCC). Molecular mechanisms underlying the activation process are currently being explored in the hope of identifying new therapeutic targets for liver fibrosis. CircRNAs have been reported to regulate HSC activation through modulating signal transduction in fibrogenic signaling pathways. Depending on which miRNAs or proteins they target, circRNAs can be either anti- or pro-fibrotic. Although most discovered circRNAs so far are mechanistically explained as miRNA sponges for their functions, some circRNAs linked to HSC activation can act as protein templates or protein sponges, but only a few studies have reported this mode of action. Given that most circRNAs do not carry multiple binding sites for a single miRNA, the sequestration of miRNAs may not be the primary function of circRNAs [207]. Future studies should explore new regulatory mechanisms of circRNAs other than miRNA sponges in HSC activation. Recent technology based on proximity labeling enzymes in couple with CRISPR/Cas allows the isolation of proteins interacting with specific RNA transcripts [208]. The identification of these proteins could yield novel functions of circRNAs in fibrogenic HSCs.

The number of newly discovered circRNAs has increased over the past years thanks to advances in nucleotide sequencing technologies. Oxford nanopore sequencing combined with circRNA identifiers using long-read sequencing data (CIRI-long) could detect additional splicing events and alternative circularization of circRNAs at a higher efficiency compared with that of Illumina RNA-sequencing methods [209]. This advanced approach might allow the identification of new circRNAs that regulate liver fibrosis. To study roles of the newly discovered circRNAs, loss-of-function and gain-of-function experiments are essential. However, current methods for such genetic manipulation have significant drawback when studying circRNAs in terms of efficiency and specificity. A recent study describes a new technique known as twister-optimized RNA for durable the overexpression (Tornado) expression system, which enables a higher rate of RNA circularization than that of traditional methods [210]. Conversely, utilizing base editors to alter single nucleotide sequences in back-splice sites could impair circRNA biogenesis and reduce its expression while avoiding off-target effects on the cognate linear mRNA [211]. These new techniques could be applied for functional analyses of circRNAs in HSC activation. Lastly, recent studies illustrate cell-to-cell communications via circRNA-containing exosomes from other liver cell types to HSCs, adding another layer of complexity in the modulation of HSC fates [124,125]. More mechanistic understanding of circRNAs in HSC activation could provide new knowledge in biology of non-coding RNA and potentially shed light on development of a new therapeutic strategy for liver fibrosis.

## Figures and Tables

**Table 1:** Overview of the circular RNA (circRNA) associated with the activation of hepatic stellate cells (HSC).

CircRNAs	Function	Pathway	Target miRNA	Target Protein	Molecular Mechanism	Role	Ref.
TGF-β signaling pathway							
CircPSD3	miRNA sponge	TGF-β signaling pathway	miR-92b-3p	Smad7	Inhibit TGF-β signal transduction by increasing the expression of Smad7, a negative regulator in TGF-β/Smad pathway	Anti-fibrotic	[113]
CircCREBBP	miRNA sponge	TGF-β signaling pathway	miR-1291	LEFTY2	Inhibit TGF-β signal transduction by increasing the expression of LEFTY2, a negative regulator in the TGF-β/Smad pathway	Anti-fibrotic	[114]
CircSCLT1	miRNA sponge	TGF-β signaling pathway	miR-223-3p	LEMD3	Inhibit TGF-β signal transduction by increasing the expression of LEMD3, a negative regulator in TGF-β/Smad pathway	Anti-fibrotic	[115]
CircMTO1	miRNA sponge miRNA sponge	TGF-β signaling pathway PI3K/Akt signaling pathway	miR-17-5p miR-181b-5p	Smad7 PTEN	Inhibit TGF-β signal transduction by increasing the expression of Smad7, a negative regulator in TGF-β/Smad pathway Inhibit PI3K/Akt signal transduction by increasing the expression of PTEN, an inhibitory signaling molecule in the PI3K/Akt pathway	Anti-fibrotic Anti-fibrotic	[116] [117]
CircBNC2	Protein template	TGF-β signaling pathway	-	ctBNC2	Reduces G2/M cell arrest of hepatocytes, which inhibits the release of pro-fibrotic factors that can activate HSCs such as TGF-β and DAMPs	Anti-fibrotic	[118]
CircPWWP2A	miRNA sponge miRNA sponge	TGF-β signaling pathway TGF-β signaling pathway	miR-203 miR-223	Fstl1 TLR4	Promote TGF-β signal transduction by increasing the expression of Fstl1, which can bind to TGF-β receptors Promote TGF-β signal transduction by suppressing the expression of BAMBI, a negative regulator in the TGF-β pathway	Pro-fibrotic Pro-fibrotic	[119] [119]
CircUbe2k	miRNA sponge	TGF-β signaling pathway	miR-149-5p	TGF-β2	Promote TGF-β signal transduction by increasing the expression of TGF-β2, a fibrogenesis mediator	Pro-fibrotic	[120]
CircTUBD1	miRNA sponge miRNA sponge	TGF-β signaling pathway TGF-β and NF-κB signaling pathway	micro-203a-3p miR-146a-5p	Smad3 TLR4	Promote TGF-β transduction by increasing the expression of Smad3 and regulating circTUBD1 biogenesis via a positive feedback loop Promote TGF-β signal transduction by suppressing the expression of BAMBI, a negative regulator in the TGF-β pathway, and promoting NF-κB signaling pathway	Pro-fibrotic Pro-fibrotic	[121] [122]
**JAK/STAT signaling pathway**							
CircCHD2	miRNA sponge	JAK/STAT signaling pathway	miR-200b-3p	HLF	Promote JAK/STAT signal transduction by increasing the expression of HLF, which can promote IL-6 expression, thus increasing IL-6/JAK/STAT3 signaling pathway	Pro-fibrotic	[123]
**PI3K/Akt signaling pathway**							
CircDIDO1	miRNA sponge	PI3K/Akt signaling pathway	miR-141-3p	PTEN	Inhibit PI3K/Akt signal transduction by increasing the expression of PTEN, an inhibitory signaling molecule in the PI3K/Akt pathway	Anti-fibrotic	[124]
CircCDK13	miRNA sponge	PI3K/Akt and NF-κB signaling pathway	miR-17-5p	KAT2B	Inhibit PI3K/Akt signal transduction by increasing the expression of PTEN, an inhibitory signaling molecule in the PI3K/Akt pathway, via inhibiting PTEN degradation	Anti-fibrotic	[125]
CircIFT80	miRNA sponge	PI3K/Akt signaling pathway	miR-155	FOXO3	Promote PI3K/Akt signal transduction by increasing FOXO3 expression	Pro-fibrotic	[126]
**Wnt/β-catenin signaling pathway**							
Circ608	miRNA sponge	Wnt/β-catenin and Hippo signaling pathway	miR-222	PINK1	Inhibit Wnt/β-catenin signal transduction by promoting the degradation of β-catenin, and inhibit Hippo signal transduction by promoting the expression of a negative regulator in the Hippo signaling pathway	Anti-fibrotic	[127]
**Notch signaling pathway**							
CircFBXW4	miRNA sponge	Notch signaling pathway	miR-18b-3p	FBXW7	Inhibit Notch signal transduction by promoting the degradation of NICD	Anti-fibrotic	[128]
**Hedgehog signaling pathway**							
CircRSF1	miRNA sponge	Hedgehog and NF-κB signaling pathway	miR-146a-5p	RAC1	Promote Hedgehog signal transduction as well as NF-κB and JNK signaling by increasing the expression of RAC1	Pro-fibrotic	[129]
**Other pathways**							
CircSCAR	Protein sponge	ROS		ATP5B	Shutting down the mPTP, which interferes with ROS release and thus inhibits HSC activation	Anti-fibrotic	[130]
CircARID1A	miRNA sponge		miR-185-3p	Col1a1	Directly promote the expression of Col1a1, a key indicator of HSC activation	Pro-fibrotic	[131]
CircSMYD4	miRNA sponge		miR-660-3p	TEP1	Possibly by maintaining telomere length, which is found to be shorten in liver diseases	Anti-fibrotic	[132]

## Data Availability

Not applicable.
